# Impact of Speed Sintering on Translucency, Opalescence and Microstructure of Dental Zirconia with a Combination of 5 mol% and 3 mol% Yttria-Stabilized Zirconia

**DOI:** 10.3390/ma17215148

**Published:** 2024-10-22

**Authors:** Mi-Hyang Cho, Hyo-Joung Seol

**Affiliations:** 1 Department of Dental Laboratory, Wonkwang Health Science University, Iksan-si 54538, Republic of Korea; milgong11@wu.ac.kr; 2 Department of Dental Materials, Dental and Life Science Institute, School of Dentistry, Pusan National University, Yangsan-si 50612, Republic of Korea

**Keywords:** zirconia, translucency, opalescence, microstructure

## Abstract

Optical characteristics and microstructure of multilayered zirconia with different yttria contents in each layer can be influenced differently with a layer after speed sintering. The layer-wise translucency and opalescence of dental zirconia (E.max, E.max ZirCAD prime; Cercon, Cercon ht ML) after conventional (control) and speed sintering were analyzed using a spectrophotometer (n = 5). Specimens were subjected to microstructural analyses (n = 2) using field-emission scanning electron microscopy (FE-SEM) and phase analyses (n = 1) using high-resolution X-ray diffraction (HRXRD) and Rietveld refinement. The translucency parameter (TP) and opalescence parameter (OP) were analyzed using a 3-way ANOVA, followed by Scheffé’s post hoc test (α = 0.05). The average grain size was analyzed using the Welch’s *t*-test and Kruskal–Wallis test, followed by the Bonferroni–Dunn post hoc test (α = 0.05). Changes to the TP and OP after speed sintering were only observed in the dentin layers. Although the TP of E.max increased (*p* < 0.05), the difference was below the 50:50% perceptibility threshold (ΔE_00_ = 0.8). The OP of E.max decreased slightly, whereas that of Cercon increased slightly (*p* < 0.05). The microstructure and phase fraction of both zirconia barely changed. Therefore, speed sintering is considered to have a negligible clinical impact on the optical characteristics and microstructure.

## 1. Introduction

Natural teeth exhibit optical translucency, allowing light to pass through to some extent, thereby creating a natural appearance [1]. In addition, natural teeth possess opalescent properties owing to the interaction of light with the intricate microstructure of the tooth incisal [2,3]. Under normal lighting, opalescence causes the incisal edge of a tooth to appear bluish [2,3]. Dental zirconia, a restorative material used to replace natural teeth, exhibits lower translucency and opalescence than natural teeth [1,2,4,5,6]. Despite this, dental zirconia is increasingly replacing its metal counterparts owing to its superior aesthetics.

Early zirconia was monochromatic and had low translucency, giving a different aesthetic to natural teeth [7]. Multilayered zirconia, typically pigmented with metal oxides to mimic the natural color of teeth [8,9], has since been developed to enhance the aesthetics of zirconia and make it suitable for monolithic restorations. An appearance similar to that of natural teeth can be achieved by multilayered zirconia, which typically comprises 4–6 mol% yttria-stabilized zirconia (4–6Y zirconia) because of the arrangement of the incisal (or enamel), transition, and dentin layers [10,11]. Although the yttria contents of all the layers are the same, the multilayered zirconia exhibits a gradual color gradient [7,11]. Pigmentation in zirconia causes a reduction in light transmission, consequently lowering its translucency [12]. In yttria-stabilized zirconia, increasing the yttria content leads to a higher proportion of cubic or yttria-rich tetragonal (*t*′) phases, endowing them with superior translucency and thus enhancing the aesthetics [11,13,14]. However, this superior translucency is achieved at the expense of the yttria-lean tetragonal (*t*) phase, which contributes to its mechanical properties via a stress-induced transformation toughening mechanism (such as the transformation from the tetragonal to monoclinic phase) [11,13,15]. Consequently, multilayered zirconia with a high yttria content exhibits significantly improved aesthetics compared to conventional 3Y-zirconia but with a significant deterioration of its mechanical properties [7,11,12,13,16].

To address the trade-off between aesthetics and mechanical properties, a novel multilayered zirconia has been developed. This new material incorporates a combination of 5Y- and 3Y-zirconia (5Y/3Y) or 5Y- and 4Y-zirconia (5Y/4Y) within a single zirconia block. In these zirconia materials, the incisal layer is primarily composed of 5Y-zirconia, and the dentin layer comprises a lower yttria (3Y or 4Y) content than the incisal layer [7,17]. Restorations made from these zirconia materials exhibit a translucency that gradually decreases from the incisal layer to the dentin layer [6,17]. However, the translucency of dentin layers made of 5Y/4Y- and 5Y-zirconia materials exhibit no statistically significant differences, despite their different yttria contents [6].

Zirconia restorations are typically fabricated using CAD/CAM (computer-aided design/manufacturing) and sintering techniques. CAD/CAM technology in dentistry offers numerous opportunities and limitations that must be carefully considered for its effective implementation. CAD/CAM technology allows for the highly accurate and precise fabrication of dental restorations, reducing the need for multiple adjustments and ensuring a better fit [18]. Additionally, CAD/CAM technology can streamline the fabrication process, reducing the time required for chairside procedures and potentially lowering costs [18]. However, CAD/CAM systems can be expensive to purchase and maintain [18]. Additionally, they require specialized technical expertise and clinical experience [18]. The conventional sintering protocol involves a long holding time at the maximum temperature and relatively low heating and cooling rates [10,19]. To address these issues, zirconia manufacturers have introduced accelerated sintering protocols that significantly reduce the sintering time. Thus, research has been conducted on the impacts of speed sintering on the optical characteristics of multilayered zirconia. Katana STML, a multilayered 5Y-zirconia with uniform composition, exhibits no change in translucency owing to speed sintering [20,21]. Another multilayered 5Y-zirconia (Cercon xt ML) shows a slight decrease in translucency in all the layers after speed sintering, along with a slight decrease in the opalescence in the dentin layer [22]. Similarly, multilayered 4Y-zirconia (Zolid Gen-X) and 6Y-zirconia (Katana UTML) exhibit slight changes in translucency and opalescence in some layers after speed sintering [23]. However, the impact of speed sintering on the optical characteristics of the recently developed multilayered 5Y/3Y-zirconia has not yet been reported. Thus, this study focused on this type of zirconia. Multilayered 5Y/3Y-zirconia exhibits a gradual color gradient, as well as different yttria contents, in each layer. Consequently, its optical characteristics can be influenced differently with the layer after speed sintering. This study aimed to investigate the impact of speed sintering on the optical characteristics and microstructure of multilayered zirconia with an incisal layer comprising 5Y-zirconia and a dentin layer comprising 3Y-zirconia (5Y/3Y-zirconia). To this end, the layer-wise optical properties of 5Y/3Y-zirconia under conventional and speed sintering conditions were compared. The null hypothesis was that speed sintering does not affect the optical characteristics or microstructure of multilayered 5Y/3Y-zirconia.

## 2. Materials and Methods

### 2.1. Sample Preparation

**Figure 1:**
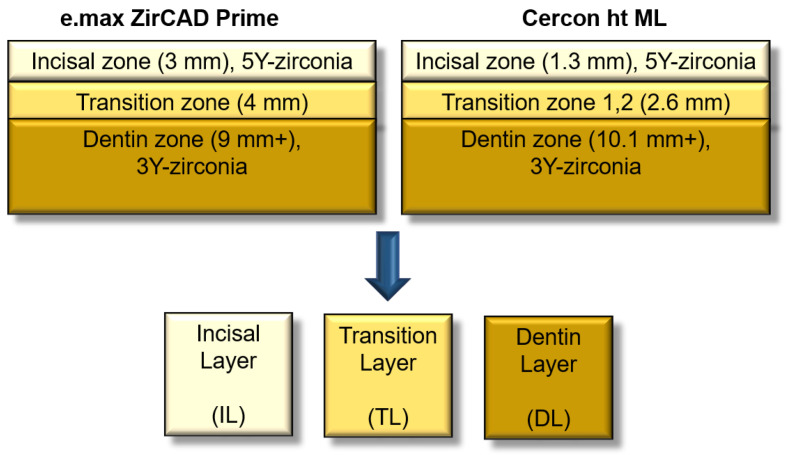
Cross-sections of the used zirconia materials (up) and specimen shape (size: 10 mm × 10 mm × 1 mm).

To analyze the optical characteristics, microstructure, and phase fraction after conventional sintering (control) and speed sintering, plate-like specimens (total sample size = 60) were prepared from each layer zone of E.max ZirCAD Prime (IvoclarVivadent, Schaan, Liechtenstein) and Cercon ht ML (Dentsply Sirona, Charlotte, NC, USA) zirconia blocks of an A2 shade (Figure 1 and Table 1). Based on the manufacturer’s specifications for the thickness of each layer zone, the specimens were carefully extracted from the central region of each layer zone by cutting and polishing (Figure 1).

Briefly, zirconia blocks were cut layer by layer using a precision cutting machine (AMC-200, Fairworks, Seoul, Republic of Korea) equipped with a diamond wheel and then sequentially polished with 800, 1200, 2000, 3000, and 5000 grit-SiC abrasive papers. After polishing, the specimen size was confirmed with micrometers (MDC-25PX, Mitutoyo, Kanagawa, Japan). The specimens were then sintered using a furnace (inLab Profire, Dentsply Sirona, Charlotte, NC, USA), according to the conventional and speed sintering protocols suggested by the manufacturer (Table 2). The sintering shrinkage was approximately 20%, and the final specimen size was 10 × 10 × 1.016 (±0.008) mm^3^.

### 2.2. Optical Characteristics Analysis

The optical characteristics of the specimens were analyzed (n = 5) in the reflectance and transmittance modes using a spectrophotometer (CM-3600d, Konica Minolta Sensing, Osaka, Japan) and Spectra-Magic software (version 2.02, Konica Minolta Sensing, Osaka, Japan). The specimens were measured three times under a CIE standard D65 light source and 2° standard observer conditions at 10 nm intervals from 360 nm to 740 nm. Transmittance mode measurements were taken by placing the specimens on the entrance hole of an integrating sphere. The translucency parameter (TP) was determined (n = 5) using the CIEDE2000 color difference (ΔE_00_) measured in the reflectance mode by placing the specimens on white (a* = −0.08, b* = 0.98, L* = 99.08) and black (a* = 0.70, b* = −0.42, L* = 9.08) backgrounds. The following formula was used for the calculations [24,25]:

$${\mathrm{TP}}_{00}(\Delta E_{00})={\left[{\left(\frac{\Delta L^{\prime }}{K_{L}S_{L}}\right)}^{2}+{\left(\frac{\Delta C^{\prime }}{K_{C}S_{C}}\right)}^{2}+{\left(\frac{\Delta H^{\prime }}{K_{H}S_{H}}\right)}^{2}+R_{T}\left(\frac{\Delta C^{\prime }}{K_{C}S_{C}}\right)\left(\frac{\Delta H^{\prime }}{K_{H}S_{H}}\right)\right]}^{1/2}$$

where Δ*L*′, Δ*C*′, and Δ*H*′ denote the differences in the lightness, chroma, and hue, respectively, detected on the white and black backgrounds; *K_L_*, *K_C_*, and *K_H_* are the parameters used to correct the discrepancies in the experimental conditions. These parameters were set to 1 [24,25]. *S_L_*, *S_C_*, and *S_H_* are the weighting functions for lightness, chroma, and hue, respectively; *R_T_* denotes a function that accounts for the interaction between chroma and hue differences in the blue region [24,25].

An opalescence parameter (OP) was obtained (n = 5) using the following formula [2]:

OP = [(a*_T_ − a*_R_)^2^ + (b*_T_ − b*_R_)^2^]^1/2^,

where a* is a red–green coordinate, and b* is a yellow–blue coordinate. The subscript T indicates the transmitted light, and the subscript R indicates the reflected light measured against a black background.

### 2.3. Microstructural Analysis

Field-emission scanning electron microscopy (FE-SEM, JSM-7200F, Jeol, Akishima, Japan) was performed on the specimens (n = 2) at room temperature (approximately 25 °C) after sputter-coating with platinum for 70–90 s. The surface morphology was observed under an acceleration voltage of 15 kV. The average grain size (equivalent diameter of the grain) was measured from the FE-SEM images using the linear intercept method [10,26] and software ImageJ version 1.53e (National Institutes of Health, Bethesda, MD, USA). A correction factor of 1.56 was used [26]. More than 700 grains were used to obtain the grain size measurements of each group.

### 2.4. Crystallographic Analysis

High-resolution X-ray diffraction (HRXRD, X’Pert3 powder, PANalytical, Amsterdam, the Netherlands) was performed (n = 1) using CuKα radiation and a Ni filter, with a tube voltage and current of 40 kV and 30 mA, respectively, in a 2θ range of 25 to 90°. The step size was set at 0.013°. Rietveld refinement of the cubic (space group: Fm3m), tetragonal (space group: P4_2_/nmc), and monoclinic (space group: P2_1_/c) phases was performed using software (Topas academic V 7.23, Bruker AXS, Karlsruhe, Germany). Crystallographic data for these phases were acquired utilizing the standard Crystallographic Information Files (CIFs) provided by Lamas and Walsoe de Reca [27] and Howard et al. [28] in the Crystallography Open Database. The refinement quality was controlled by keeping the Rwp values below 6.5%. The Y_2_O_3_ content (mol %) of the tetragonal phase was determined using the obtained lattice parameters (*a*, *c*) using the following equations [29,30]:

$${\mathrm{YO}}_{1.5}\;(\mathrm{mol}\%)=\frac{1.0223-c/a\sqrt{2}}{0.001319}$$

$$Y_{2}O_{3}\;(\mathrm{mol}\%)=\frac{{YO}_{1.5}/100}{2-{YO}_{1.5}/100}\times 100$$

### 2.5. Statistical Analysis

The experimental results were statistically analyzed (α = 0.05) using the statistical program (Statistical Product and Service Solutions 25.0, IBM Co., Armonk, NY, USA). The TP and OP were analyzed using a 3-way ANOVA, followed by Scheffé’s post hoc test. The Δa* (a*_T_ − a*_R_) and Δb* (b*_T_ − b*_R_) between the transmitted color and reflected color were analyzed using a generalized linear model. Grain size was analyzed using the Welch’s *t*-test and Kruskal–Wallis test (with the Bonferroni–Dunn post hoc test).

## 3. Results

### 3.1. Optical Characteristics

**Figure 2:**
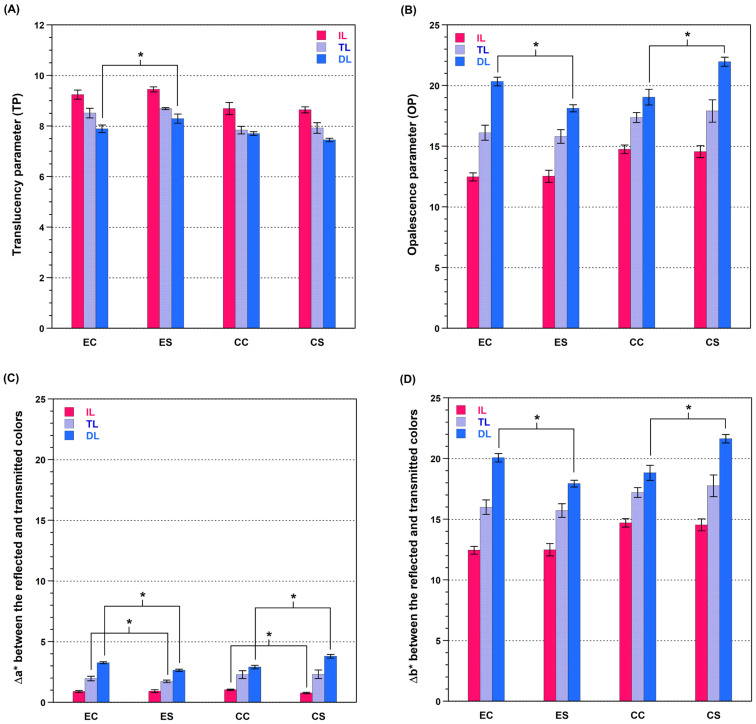
Translucency, opalescence, and Δa* and Δb* of specimens. (**A**) Translucency parameter of conventional- and speed-sintered zirconia. (**B**) Opalescence parameter of conventional- and speed-sintered zirconia. (**C**) Δa* of conventional- and speed-sintered zirconia. (**D**) Δb* of conventional- and speed-sintered zirconia. (IL, incisal layer; TL, transition layer; DL, dentin layer; EC, conventionally sintered E.max; ES, speed sintered E.max; CC, conventionally sintered Cercon; CS, speed sintered Cercon). The asterisk-marked lines indicate the differences between the groups were statistically significant (*p* < 0.05).

As shown in Table 3, the three-way ANOVA results indicate that the material, sintering speed, and layer had a significant influence on the TP and that there were interactions between material and speed; material and layer; and material, speed, and layer. Figure 2A shows the TP results for each group. For Cercon, TP was not affected by speed sintering (*p* > 0.05). Although E.max showed the same trend, the TP in the dentin layer increased after speed sintering (*p* < 0.05). The TP value increased from the dentin layer to the incisal layer irrespective of the sintering speed for both zirconia materials (*p* < 0.05).

As shown in Table 4, the three-way ANOVA results of OP indicate interactions between material and layer material and speed; and material, speed, and layer. In the dentin layer (Figure 2B), the OP of E.max decreased, whereas that of Cercon increased after speed sintering (*p* < 0.05). Both zirconia materials exhibited an increase in their OP values from the incisal layer to the dentin layer irrespective of the sintering speed (*p* < 0.05). The OP values were obtained from Δa* and Δb* between the transmitted color and reflected color. Based on this, the changes in Δa* and Δb* owing to speed sintering were analyzed to understand how speed sintering affects opalescence. In both zirconia materials (Figure 2C,D), Δa* showed significant differences for the various layers owing to the speed sintering, but the numerical differences were negligible. In both zirconia materials, Δb* of only the dentin layer was affected by the speed sintering, as it was in the OP, resulting in a decrease of Δb* in E.max and an increase of Δb* in Cercon (*p* < 0.05).

### 3.2. Microstructure

**Figure 3:**
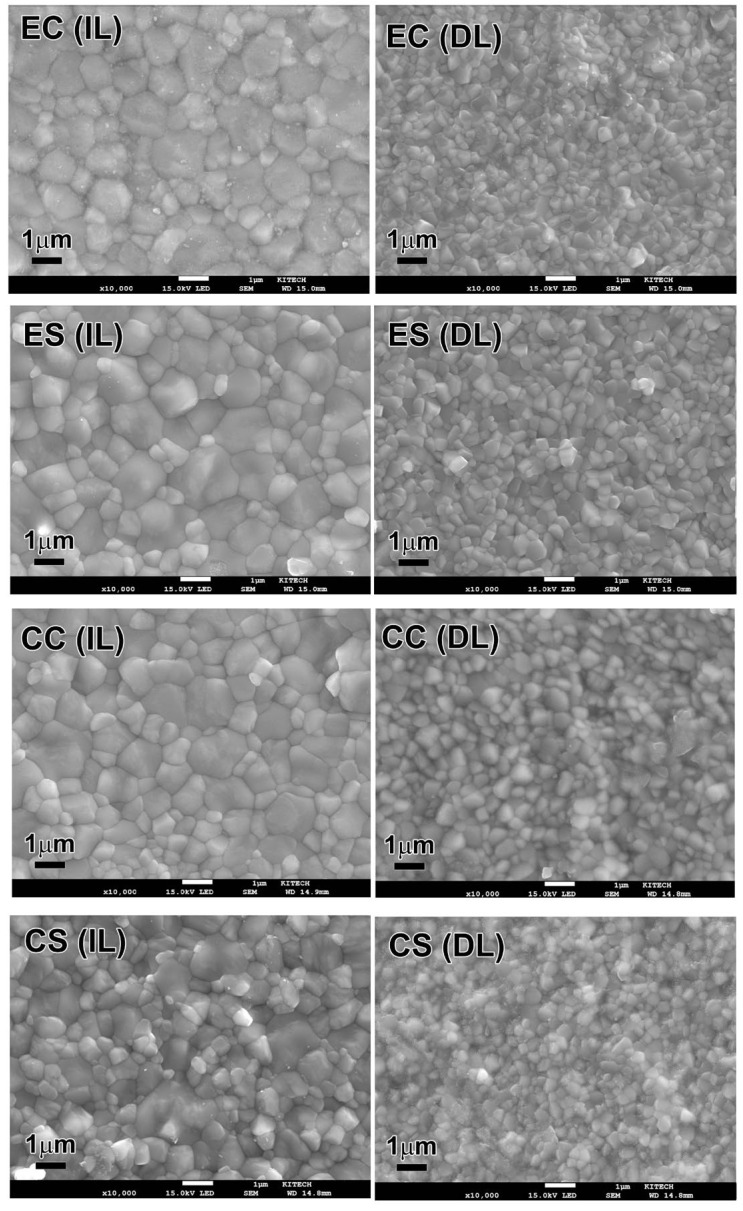
Microstructure of conventional- and speed-sintered zirconia (×10,000). IL, incisal layer; DL, dentin layer; EC, conventionally sintered E.max; ES, speed sintered E.max; CC, conventionally sintered Cercon; CS, speed sintered Cercon.

Both zirconia materials exhibited equiaxed microstructures, regardless of the sintering speed (Figure 3). The crystal grains in the dentin layer were relatively smaller than those in the incisal layer. Table 5 lists the average grain sizes (equivalent diameter of the grain) for each group. The grain size of E.max was not affected by the speed sintering (*p* > 0.05), whereas that of Cercon decreased slightly (*p* < 0.05). The average grain sizes for both zirconia materials were approximately 1 μm and 0.5 μm in the incisal and dentin layers, respectively.

### 3.3. Crystal Structure

The phase fractions of both zirconia materials barely changed by speed sintering (Table 6 and Table S1). Both zirconia materials comprised yttria-lean tetragonal (*t*-phase) and yttria-rich tetragonal (*t*′-phase) phases, with no monoclinic phase. The yttria-lean *t*-phase contents of both zirconia materials were higher in the dentin layers than that in the incisal layers. The axial ratio of the *t*- and *t*′-phases were approximately 1.015 and 1.005, respectively, for both types of zirconia. The yttria content in the *t*′-phase was higher (6.8 to 7.4 mol%) than that in the *t*-phase (2.4 to 2.8 mol%).

## 4. Discussion

In this in vitro study, the null hypothesis that speed sintering does not affect the optical characteristics and microstructure of 5Y/3Y-zirconia is partially rejected. For Cercon, TP was not affected by speed sintering (*p* > 0.05). E.max exhibited a similar trend, except for the dentin layer, where the speed sintering led to an increase in the TP (*p* < 0.05). However, the difference in the TP values was 0.4, which is below the 50:50% perceptibility threshold of CIEDE2000 (ΔE_00_ = 0.8) [31]. Thus, the change was not considered clinically significant. Similarly, speed sintering has been reported to increase the TP in the dentin layer of multilayered 4Y-zirconia and decrease the TP in the transition layer of multilayered 6Y-zirconia [23]. However, these differences were minimal and fell below the 50:50% perceptibility threshold [23]. In addition, multilayered 5Y-zirconia exhibited a minor decrease in translucency in all the layers after speed sintering [22]. In the study, both Lava Esthetic, a conventional sintering material, and Cercon xt ML, a speed sintering material, experienced a slight decrease in translucency when subjected to speed sintering [22]. However, the difference in TP was imperceptible [22].

The addition of stabilizers, such as yttria, to zirconia increases its translucency [11,13,15]. The TP of both zirconia materials decreased from the incisal layer (5Y) to the dentin layer (3Y), irrespective of the sintering speed (*p* < 0.05). Similar results have been reported for 5Y/4Y- and 5Y/3Y-zirconia under conventional sintering [6,17] and even for 4Y-zirconia and 6Y-zirconia with uniform composition [23]. According to the literature, pigmentation significantly reduces the light transmission of zirconia, thereby lowering its translucency [12]. Therefore, the decrease in the TP value from the incisal to the dentin layer was thought to be caused primarily by the pigmentation gradient toward the dentin layer in the 5Y/3Y-zirconia, followed by the yttria gradient. In this study, the obtained TP values were approximately 9 in all the specimens of incisal layer (enamel layer). This value is similar to those for 4Y-zirconia after speed sintering (8.6) and those for 6Y-zirconia after speed sintering (9.0) [23].

A change in the OP value after speed sintering was observed only in the dentin layer of both zirconia materials. Speed sintering slightly decreased the OP value of E.max and slightly increased that of Cercon (*p* < 0.05). A slight decrease in the OP value after speed sintering has also been reported for multilayered 4Y- and 6Y-zirconia [23]. In the study, speed sintering significantly reduced OP in the dentin layer and incisal layer of 6Y-zirconia [23]. In 4Y-zirconia, OP decreased after speed sintering in the dentin layer and transition layer [23].

For 5Y-zirconia designed for speed sintering, the OP values of the dentin and transition layers decreased, whereas the OP value decreased in all the layers in 5Y-zirconia that was not designed for speed sintering [22]. The OP value of the human incisal is in the range of 19.8 to 27.63 at a thickness of 1 mm [2]. In this study, the OP value of the incisal layer (12.47 to 14.74) was lower than that of human incisal. The dentin layer (3Y) exhibited a higher OP value (18.12 to 21.96) than the incisal layer (*p* < 0.05). For 6Y-zirconia, the OP values of the dentin layer after speed and conventional sintering were approximately 18 and 19, respectively [23]. The OP values of the dentin layer of 4Y-zirconia after speed and conventional sintering were reported to be approximately 21 [23]. These OP values were similar, even when the yttria content of the dentin layer varied from 3 mol% to 6 mol%. Thus, the OP values did not change significantly with an increase in the yttria content. Instead, the change in the OP value was consistent with the change in Δb* between the reflected color and transmitted color after speed sintering. An extremely high correlation between the OP value and Δb* has also been reported in human and bovine incisal [2]. The difference in the changes in the OP (increase or decrease) of the two zirconia samples seemed to result from the differences in the type and amount of pigment contained in the zirconia. In this study, the TP decreased, whereas the OP increased from the incisal layer to the dentin layer for both zirconia materials irrespective of the sintering speed (*p* < 0.05). These results have previously been reported for multilayered zirconia with a constant yttria content [23]. Therefore, the decrease in the TP, along with the increase in the OP from the incisal layer to the dentin layer in 5Y/3Y zirconia, is mainly caused by the pigmentation gradient, with a minor effect induced by different yttria contents in each layer.

Microstructural observations revealed that the grain size of E.max was unaffected by the speed sintering (*p* > 0.05). The grain size of Cercon decreased by speed sintering (*p* < 0.05); however, the difference in values was very small. Similarly, the grain size of the multilayered zirconia with a constant yttria content has been reported to exhibit no or minimal decrease by the speed sintering [22,23]. In this study, the average grain sizes for both zirconia materials were approximately 1 μm and 0.5 μm in the incisal and dentin layers, respectively. These values are within the average grain size range obtained from conventionally sintered 5Y- and 3Y-zirconia materials, respectively [22,30].

In addition to the grain size, the phase fraction also affects the translucency of zirconia [30]. Previously, the presence of a cubic phase in 4Y- to 6Y-zirconia was widely accepted [10,11,21], but recent studies have indicated that the yttria-rich *t*′-phase with an axial ratio similar to that of the cubic phase exists in 4Y- to 6Y-zirconia instead of the cubic phase [8,29]. The yttria-rich *t*′-phase has a lower tetragonality (axial ratio) than the yttria-lean *t*-phase, thus reducing the light scattering caused by birefringence [8,14,29]. Therefore, an increase in the *t*′-phase contributes to an increase in the translucency of zirconia [14]. Both zirconia materials revealed the presence of a higher *t*-phase in the dentin layer than that in the incisal layer. The phase fraction was barely altered after speed sintering for both zirconia materials. Therefore, the changes in the TP and OP observed after speed sintering were unlikely to be caused by the phase changes. The obtained phase fraction in each layer and tetragonality (axial ratio) of the yttria-rich *t*′-phase and yttria-lean *t*-phase were within the range of values reported for 5Y- and 3Y-zirconia [8,14].

Dental zirconia is usually pigmented with metal oxides to mimic the natural color of teeth [8]. In multilayered zirconia, the dentin layer typically contains more pigment than the incisal layer [8,9]. Consequently, the optical properties of the dentin layer can be more sensitive to the influence of pigmentation than those of the incisal layer. In this study, changes in the TP and OP were observed only in the dentin layer after speed sintering. These changes, however, were minimal. Therefore, speed sintering is considered to have a negligible clinical impact on the optical characteristics and microstructure of tested 5Y/3Y-zirconia.

The emergence of advanced materials in digital dentistry, such as zirconia, offers exciting possibilities for improving the strength, durability, and aesthetics of dental restorations [10]. As the durability of dental restorations hinges not only on material properties but also on cementation techniques [32,33], further research on cementation techniques is needed to fully understand their long-term clinical performance. The limitation of this investigation is that only A2-shade zirconia and a one-speed sintering protocol were applied. To further analyze the impact of speed sintering on the optical characteristics of multilayered 5Y/3Y-zirconia, various sintering protocols and zirconia with a wider variety of colors need to be included in the analysis. In addition, as many commercial products as possible should be covered to obtain a complete understanding of the newly developed multilayered 5Y/3Y-zirconia.

## 5. Conclusions

The speed sintering process caused minimal alterations in translucency and opalescence of the tested 5Y/3Y zirconia. These changes were limited to the dentin layer, suggesting they likely have no noticeable effect on clinical applications. To thoroughly evaluate the multilayered 5Y/3Y-zirconia, it will be important to further study the mechanical properties of zirconia before and after aging and fatigue properties to obtain a complete understanding of their long-term viability.

## Figures and Tables

**Table 1:** Chemical composition of the used materials.

Material	Type	Chemical Composition (wt%)			
		ZrO_2_	HfO_2_	Y_2_O_3_	Al_2_O_3_ + SiO_2_ + Others
E.max ZirCAD Prime	5Y (Incisal zone)	>88	≤5	≤7	≤2.5
	3Y (Dentin zone)	88–95.5	≤5	>4.5	≤2.5
Cercon ht ML	5Y (Incisal zone)	>86	<3	9	<2
	3Y (Dentin zone)	>90	<3	5	<2

**Table 2:** Sintering protocols of the used zirconia materials.

Material	Sintering Speed	Code	Stage	Heating and Cooling Rate (°C/min)	Temp. (°C)	Holding Time (min)
E.max ZirCAD Prime	Conventional	EC	1	10	900	30
			2	3	1500	120
			3	10	900	0
			4	8	300	0
	Speed	ES	1	60	1000	10
			2	3	1530	60
			3	50	1100	0
			4	60	100	0
Cercon ht ML	Conventional	CC	1	22	880	0
			2	11	1500	135
			3	99	300	0
			4	25	50	0
	Speed	CS	1	23	1100	0
			2	14	1350	0
			3	7	1520	35
			4	99	750	0

EC, conventionally sintered E.max; ES, speed sintered E.max; CC, conventionally sintered Cercon; CS, speed sintered Cercon.

**Table 3:** Results of a 3-way ANOVA of the TP.

Source of Variation	Type III Sum of Squares	Degree of Freedom	Mean Square	F-Statistic	*p*-Value
Corrected model	20.932 ^a^	11	1.903	81.144	**<** **0** **.001**
Intercept	4195.550	1	4195.550	178,908.349	**<** **0** **.001**
Material	6.036	1	6.036	257.376	**<** **0** **.001**
Speed	0.151	1	0.151	6.439	**0** **.014**
Layer	14.040	2	7.020	299.342	**<** **0** **.001**
Material × Speed	0.389	1	0.389	16.580	**<** **0** **.001**
Material × Layer	0.140	2	0.070	2.984	0.060
Speed × Layer	0.005	2	0.002	0.105	0.901
Material × Speed × Layer	0.172	2	0.086	3.662	**0** **.033**
Error	1.126	48	0.023		
Total	4217.607	60			
Corrected total	22.057	59			

^a^ R squared = 0.949 (Adjusted R squared = 0.937).

**Table 4:** Results of a 3-way ANOVA of the OP.

Source of Variation	Type III Sum of Squares	Degree of Freedom	Mean Square	F-Statistic	*p*-Value
Corrected model	476.018 ^a^	11	43.274	163.016	**<0** **.001**
Intercept	16,816.847	1	16,816.847	63,349.639	**<0** **.001**
Material	43.380	1	43.380	163.413	**<0** **.001**
Speed	0.265	1	0.265	0.998	0.323
Layer	396.202	2	198.101	746.253	**<0** **.001**
Material × Speed	13.713	1	13.713	51.657	**<0** **.001**
Material × Layer	1.892	2	0.946	3.564	**0** **.036**
Speed × Layer	0.458	2	0.229	0.863	0.428
Material × Speed × Layer	20.108	2	10.054	37.874	**<0** **.001**
Error	12.742	48	0.265		
Total	17,305.607	60			
Corrected total	488.760	59			

^a^ R squared = 0.974 (Adjusted R squared = 0.968).

**Table 5:** Average grain size of each specimen (M ± SD).

Code/Layer	IL	DL
**EC**	1.19 ^BCb^ (0.06)	0.48 ^Aa^ (0.03)
**ES**	1.31 ^Cb^ (0.10)	0.51 ^ABa^ (0.03)
**CC**	1.15 ^Bb^ (0.07)	0.53 ^Ba^ (0.02)
**CS**	1.01 ^Ab^ (0.10)	0.47 ^Aa^ (0.04)

There is no significant difference among the groups listed vertically when the letters are the same uppercase. There is no significant difference between the two layers (IL and DL) when the letters are the same lowercase (IL, incisal layer; DL, dentin layer; EC, conventionally sintered E.max; ES, speed sintered E.max; CC, conventionally sintered Cercon; CS, speed sintered Cercon).

**Table 6:** Zirconia phase characterization by Rietveld analysis.

Layer		IL				DL			
Phase	Code	EC	ES	CC	CS	EC	ES	CC	CS
**Yttria-lean (*t*)**	wt%	37.28 (0.33)	39.09 (0.50)	36.01 (0.58)	39.48 (0.27)	76.24 (0.40)	75.94 (0.46)	74.22 (0.50)	76.99 (0.23)
	Axial ratio	1.0151	1.0153	1.0157	1.0155	1.0161	1.0157	1.0156	1.0150
	Y_2_O_3_ (mol%)	2.81	2.72	2.58	2.63	2.43	2.56	2.60	2.83
**Yttria-rich (*t*′)**	wt%	62.72 (0.33)	60.91 (0.50)	63.99 (0.58)	60.52 (0.27)	23.76 (0.40)	24.06 (0.46)	25.78 (0.50)	23.01 (0.23)
	Axial ratio	1.0052	1.0055	1.0051	1.0056	1.0041	1.0049	1.0055	1.0052
	Y_2_O_3_ (mol%)	6.93	6.81	6.99	6.77	7.41	7.06	6.82	6.93

IL, incisal layer; DL, dentin layer; EC, conventionally sintered E.max; ES, speed sintered E.max; CC, conventionally sintered Cercon; CS, speed sintered Cercon. The numbers in parentheses represent error values.

## Data Availability

The original contributions presented in the study are included in the article, further inquiries can be directed to the corresponding author.

## Supplementary Materials

The following supporting information can be downloaded at: https://www.mdpi.com/article/10.3390/ma17215148/s1, Table S1: XRD data.
